# Characterisation of redox states of metal–organic frameworks by growth on modified thin-film electrodes†
†Electronic supplementary information (ESI) available. See DOI: 10.1039/c8sc00803e


**DOI:** 10.1039/c8sc00803e

**Published:** 2018-06-04

**Authors:** Tamoghna Mitra, Florian Moreau, Adam Nevin, Carlo U. Perotto, Alex Summerfield, E. Stephen Davies, Elizabeth A. Gibson, Timothy L. Easun, Martin Schröder

**Affiliations:** a School of Chemistry , The University of Nottingham , University Park , Nottingham , NG7 2RD , UK . Email: M.Schroder@manchester.ac.uk; b Department of Chemistry , The University of Liverpool , Crown Street , Liverpool , L69 7ZD , UK; c School of Chemistry , The University of Manchester , Oxford Road , Manchester M13 9PL , UK; d School of Physics and Astronomy , The University of Nottingham , University Park , Nottingham , NG7 2RD , UK; e School of Chemistry , Newcastle University , Bedson Building , Newcastle upon Tyne NE1 7RU , UK; f School of Chemistry , Cardiff University , Park Place , Cardiff , CF10 3AT , UK . Email: EasunTL@cardiff.ac.uk

## Abstract

The application of metal–organic framework (MOF) materials in electrochemical and electrochromic devices remains rare. One of the main reasons for this is the inability to readily access their detailed electrochemistry. The inherent insolubility of these materials does not allow interrogation by traditional solution-based electrochemical or spectroscopic methods. In this study, we report a straightforward alternative approach to the spectroelectrochemical study of MOFs. We have used two systems as exemplars in this study, MFM-186 and MFM-180. The method involves chemical modification of a working electrode to attach MOF materials without using corrosive reagents such as inorganic acids or bases which otherwise could limit their application in device development. MFM-186 demonstrates the formation of a stable radical species [MFM-186]˙^+^ on electrochemical oxidation, and this has been characterised by electrochemical, spectroelectrochemical and EPR spectroscopic techniques coupled to DFT analysis.

## Abstract 2

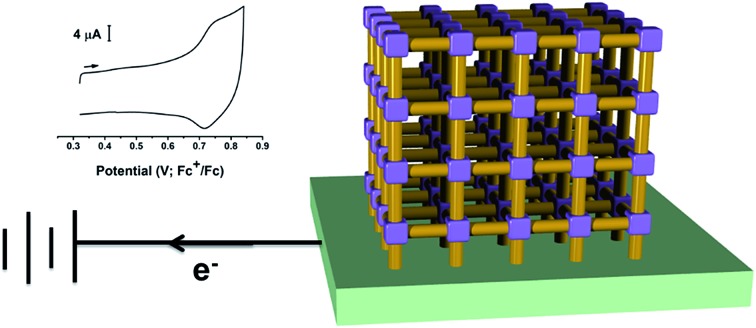
Two different SURMOF films have been grown on a transparent conducting surface for spectro-electrochemical characterisation of radical species.

## Introduction

The last decade has seen enormous progress in the field of self-assembled porous materials,^1^ particularly of crystalline, microporous metal–organic framework (MOF) materials.^2^,^3^ The crystalline nature of MOF materials enables their study by X-ray crystallographic methods,^4^ and their high porosity and internal surface areas are properties which have been exploited for storage, separation and mass transfer^5^–^9^ applications.^10^,^11^ More recently MOFs have been considered in electrocatalysis^12^–^14^ and in electrochromic devices,^15^,^16^ and there are also a few reports on light-induced modification of both MOF structures and redox properties.^17^–^22^ However, there remain significant obstacles in this area particularly with regard to the insulating nature of many framework systems, and detailed studies into the redox activity of MOFs remain scarce due mainly to the insolubility of MOF materials and the lack of general, straightforward methods to study MOFs in their non-native redox states.^23^–^25^ One way to address this scarcity is to combine reaction-oriented bulk electrolysis of MOFs with species-focused *in situ* spectroscopic methods such as UV-Vis or IR absorption spectroscopies. A key challenge here is to prepare an optically transparent electrode (OTE) modified/coated with a thin film of MOF to allow spectroelectrochemical characterisation of redox processes leading potentially to exploitation of electrochromic effects.

Attempts have been made to elucidate the spectral properties of MOFs in their redox accessible states using *in situ* UV-Vis-NIR spectroelectrochemical techniques.^26^ One approach is to affix crystalline MOF particles onto an optically transparent indium-doped tin oxide electrode (ITO) using an electrolyte intercalated polyvinylchloride as a form of mechanical ‘glue’.^26^ Though the simplicity of this route makes it a powerful method for studying the redox properties of MOFs, the reliability of contact between conducting surface and mechanically affixed MOF crystals can be an issue. Solvothermal synthesis of a redox active and optically transparent thin film of a water-stable pyrazolate-based MOF bound to a conducting fluorine-doped tin oxide (FTO) coated glass has been reported.^16^ The key feature of this route is the strong adhesion of the pyrazolate-based crystals to the FTO surface. Unfortunately, most MOF crystals do not tend to adhere strongly to surfaces without external modification, making current methods applicable to only specific MOFs. Moreover, many MOF syntheses require the presence of corrosive acid or base which can harm the conductive surface. We, therefore, devised an alternative path to synthesise a thin film of MOF on a transparent conducting surface. Synthesis of surface mounted MOF films, so-called SURMOF, has been reported whereby solutions of structural components are deposited sequentially onto non-transparent functionalized gold substrates *via* a ‘layer-by-layer’ (LBL) growth method.^27^–^29^ The resulting films are often described as ultra-thin, oriented, and thickness adjustable, although the uniformity of the SURMOF layers and controllability of the growth are not always entirely clear cut.^30^ Despite this, the generality and simplicity of this sequential deposition approach has allowed us to synthesis a MOF film onto a modified electrode. We report herein the synthesis and electrochemical properties of MOF films containing redox-active organic linkers on the conducting platforms of ITO-glass and carbon paper.

**Fig. 1:**
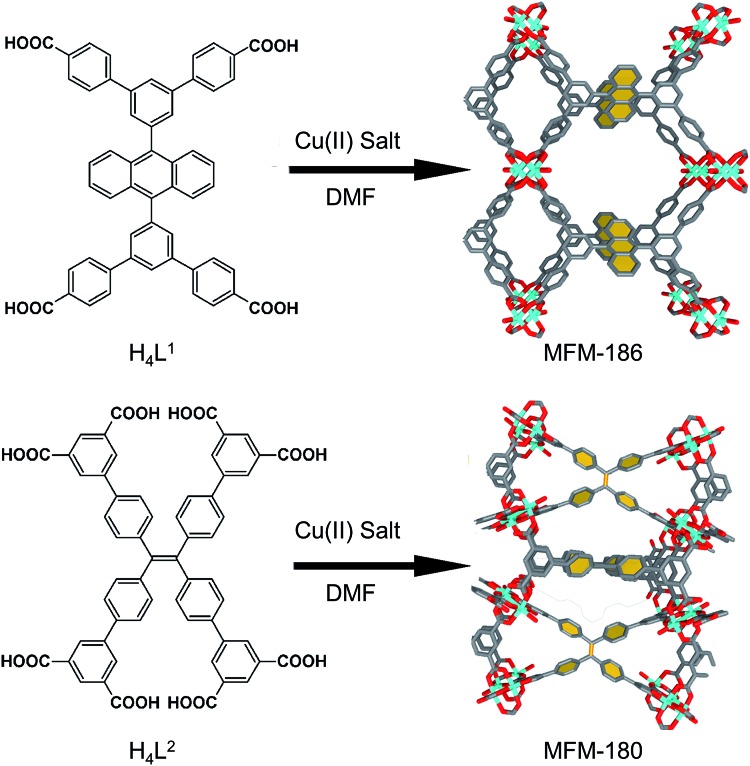
Synthesis and structure of MFM-186 (top) and MFM-180 (bottom). The redox-active core of these two MOFs are highlighted in yellow. Colour code: C: dark gray, Cu: blue, O: red.

We have long been concerned with the development of modular syntheses for Cu(ii) paddlewheel MOFs with poly-aromatic, highly conjugated organic linkers at their core.^31^,^32^ One such family of frameworks are based on Cu(ii) paddlewheel nodes with octa- and tetra-carboxylate ligands.^33^ For the current study, we chose frameworks containing redox active 9,10-phenylanthracene (H_4_L^1^) in MFM-186 (ref. 32) and tetraphenylethylene (H_8_L^2^) in MFM-180 (also known as PCN-922)^33^,^34^ (Fig. 1). Both MOFs can be synthesised as crystalline powders by heating Cu(ii) salts with the organic ligand in *N*,*N*-dimethylformamide (DMF) at 80 °C in a sealed tube. MFM-186 crystallises in orthorhombic space group *Imma*, while MFM-180 crystallises in tetragonal space group *I*4[combining macron]2*m*. Both framework systems have paddlewheel units linked by tetracarboxylate (MFM-186)^32^ or octacarboxylate (MFM-180)^33^,^34^ linkers to form porous 3D lattices.

## Results and discussion

### Synthesis of modified electrode

**Fig. 2:**
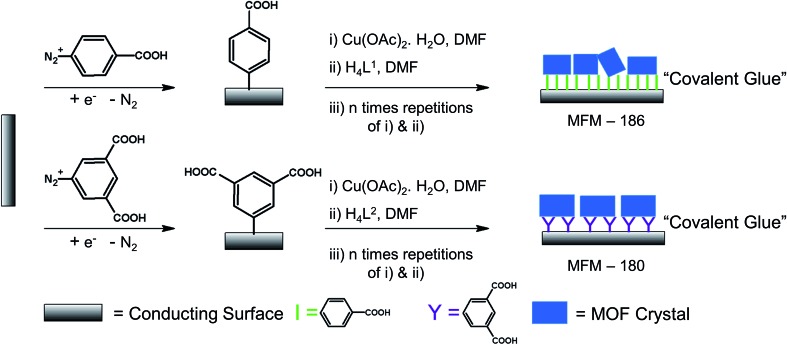
Illustration of chemical modification of conducting surface with crystalline MOF films.

To grow a film of each MOF on ITO-coated glass or carbon paper the conducting platform needs to be functionalized. For this purpose, electrode surfaces were coated with a monolayer of 4-phenyl carboxylic or 3,5-phenyl dicarboxylic acid *via* electrodeposition of 4-carboxylic- or 3,5-dicarboxlic-phenyl diazonium cations (Fig. S1†).^35^ These diazonium cations were chosen to match the end group of the corresponding ligand. Thus, to grow MFM-186 the 4-carboxylic phenyl diazonium salt was used, and for MFM-180 the 3,5-dicarboxlic phenyl diazonium salt was chosen (Fig. 2). The efficient functionalization of the surface can be monitored by measuring the conductivity of the surface, before and after functionalization. Formation of an aryl-monolayer blocks the surface and slows down the electron transfer processes between the electrolyte solution and the underlying conductive surface. This blocking effect is demonstrated for our samples by electrochemical impedance spectroscopic measurements. We found that the formation of MOF could be readily achieved by sequentially immersing the functionalized surface in DMF solutions of metal salts and ligand, rather than by the normal solvothermal synthesis in a sealed vessel. Between each immersion, the surface was washed with DMF to remove unreacted reagents. These immersion cycles were repeated (10, 15, 20 or 50 times) to obtain films of different thicknesses. The immobilisation of these films was demonstrated by their resilience to multiple washings in a range of organic solvents (DMF, CH_2_Cl_2_, CH_3_CN) or to sonication in CH_2_Cl_2_. We attribute this resilience to the covalent linkage between MOF and surface afforded by the electrodeposited aryl monolayer acting as “covalent glue”.

As anticipated,^36^,^37^ PXRD of these films confirms their crystalline nature, with the peak intensities strongly suggesting a high degree of preferred orientation within the films (Fig. S7†). Unsurprisingly, atomic force microscopy (AFM) indicates their thickness increases with increasing numbers of immersion cycles.^38^,^39^ However, AFM images indicate that these films consist of aggregates of nano crystals rather than the coverage that might be expected from pure epitaxial type growth (Fig. S6†). This is unsurprising given the relatively rough surfaces afforded by ITO and carbon paper electrodes. As shown by AFM (Fig. S6†) the MFM-180 film is made of continuous packing of MOF crystallites. MFM-186, however, forms a discontinuous film made up of isolated islands of agglomerated crystallites.

### Electrochemical and DFT analysis

**Fig. 3:**
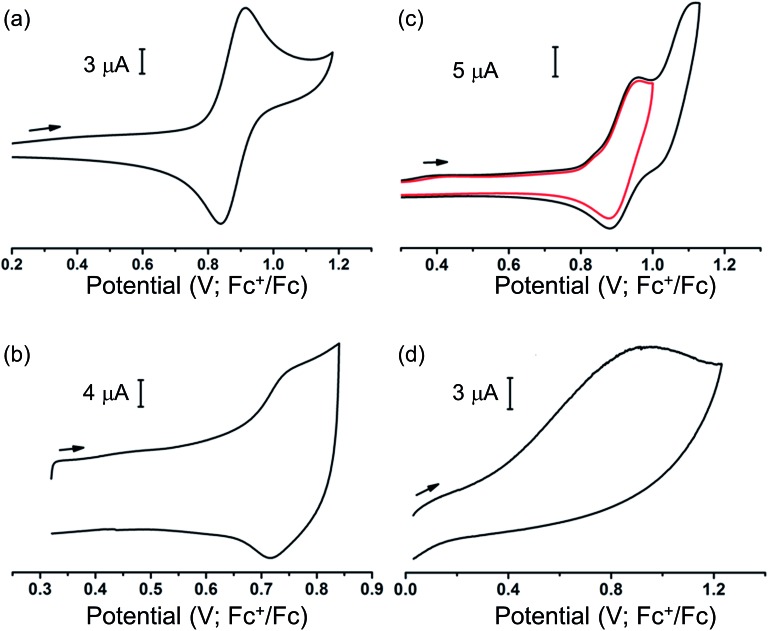
Cyclic voltammograms of (a) [Et_4_L^1^], (b) thin film of MFM-186 on carbon paper, (c) [Et_8_L^2^] and (d) thin film of MFM-180 on carbon paper, recorded at ambient temp in 0.4 M [*^n^*Bu_4_N][BF_4_] solution in CH_2_Cl_2_.

Our ability to grow thin films of highly oriented, covalently-bound thin MOF films encouraged us to explore the electrochemical behaviour of the films. Cyclic voltammetry (CV) measurements were carried out in CH_2_Cl_2_ solutions containing [^*n*^Bu_4_N][BF_4_] (0.4 M) as supporting electrolyte using a three-electrode electrochemical cell comprising of a platinum wire as the counter electrode, an Ag/AgCl (sat. KCl) as reference electrode and the MOF films grown on carbon paper acting as the working electrode. For cyclic voltammetry, the films grown on carbon paper were used as working electrodes primarily due to the superior conductivity of the carbon paper over ITO-coated glass. Conversely, the ITO-based films were used as working electrodes for subsequent spectroelectrochemical studies to exploit the transparent nature of the ITO-coated glass. Prior to any electrochemical study, the guest DMF within the framework pores was exchanged with CH_2_Cl_2_. The films were conditioned by soaking in the electrolyte solution for 24–48 h to allow maximum electrolyte diffusion into the pore structure prior to the electrochemical experiment. The cyclic voltammograms of each MOF film and those of solutions of ethyl esters of their corresponding ligands in CH_2_Cl_2_ are shown in Fig. 3. Each film exhibited well-defined redox behaviour consistent with oxidation of MOF frameworks, described further below.

The MFM-186 film showed a reversible process at *E*_1/2_ = 0.75 V (*vs.* Fc^+^/Fc). The observed peak separations (Δ*E* = 28 mV at 5 mV s^–1^ scan rate) and the relationship between the peak currents and scan rate (*i* ∝ scan rate^0.82^) suggests that the system is neither completely diffusion controlled (ideally Δ*E* = 59 mV, *i* ∝ scan rate^0.5^) nor surface-confined (ideally Δ*E* = 0 mV, *i* ∝ scan rate). The effects of mass transfer limitations within the film due to hindered diffusion, as might be expected for porous, non-conductive films,^40^ are also visible (see Fig. S8† and related discussion for additional details). CV measurements in solution for the ethyl ester Et_4_L^1^ using a glassy carbon electrode showed a reversible oxidation process at *E*_1/2_ = 0.88 V (Fig. 3). This was shown to be a one electron oxidation process by coulometry. The slight cathodic shift of the oxidation process in the MOF film relative to Et_4_L^1^ is consistent with greater negative charge density on the deprotonated ligand in the MOF framework. Notably, the MFM-186 film also showed excellent stability to electrochemical cycling with no decrease in the peak current observed over multiple cycles at scan rates between 2 and 10 mV s^–1^. In contrast, the octaester Et_8_L^2^ shows two oxidation processes at *E*_1/2_ = 0.92 V and *E*ap = 1.11 V, the latter not demonstrating a return wave in the return cycle at the scan rates employed. We attribute the first process to the formation of a tetra-phenyl ethylene based radical ion.^41^ This behaviour differs from that of MFM-180 film which displays only an irreversible process (*E*ap = 0.92 V) (Table 1).

**Table 1:** Summary of cyclic voltammetric data

	Et_4_L^1^	MFM-186	Et_8_L^2^	MFM-180
*E*_1/2_ ^*a*^ (V)	0.88	0.75	0.92	—
*E* a p ^*a*^ (V)	—	—	1.11	0.92

^*a*^ *E*_1/2_ = (*E*ap + *E*cp)/2, *E*ap = peak anodic potential; *E*cp = peak cathodic potential.

### Bulk electrochemical and DFT analysis of MFM-186

**Fig. 4:**
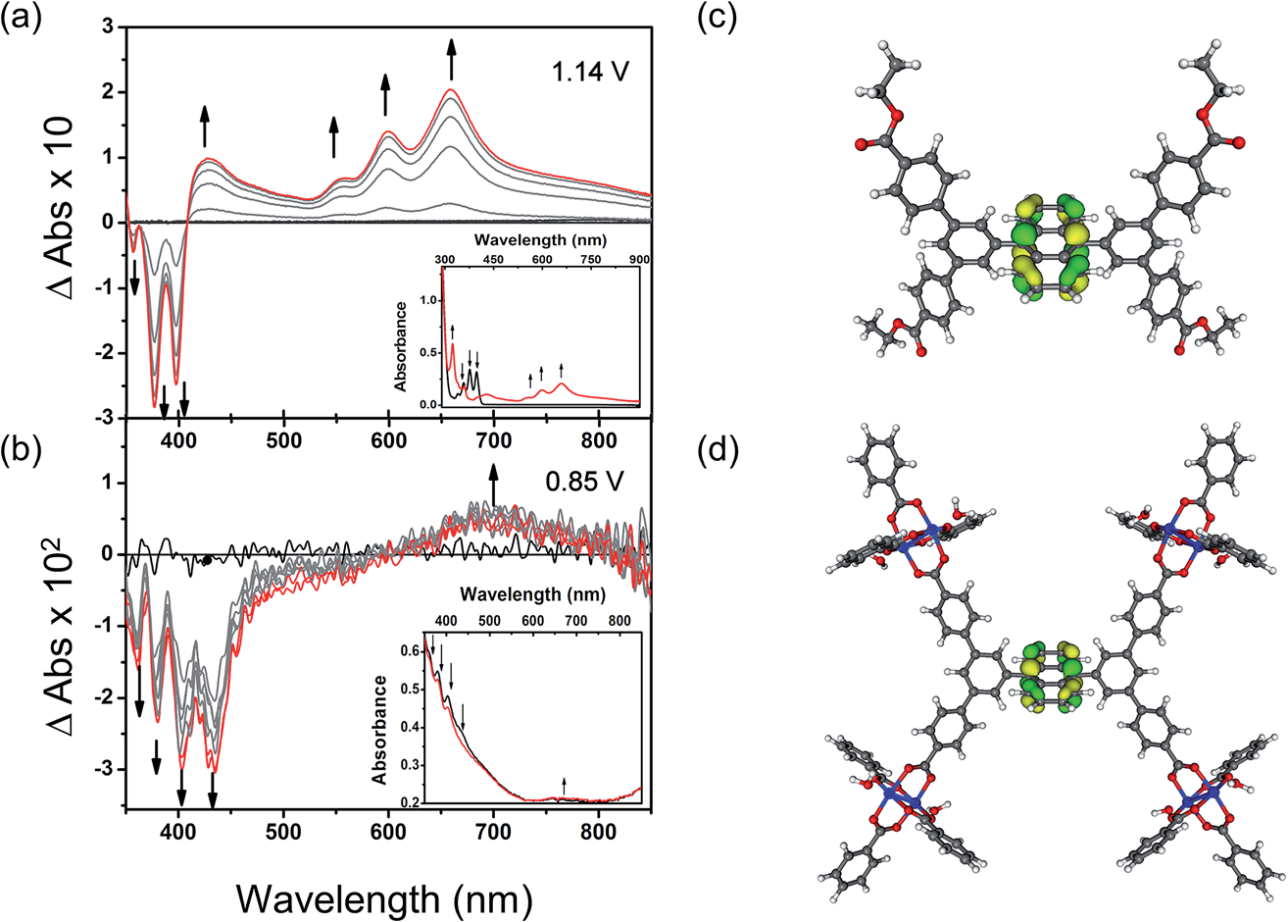
Time-dependent difference spectra of electrochemically oxidised (a) Et_4_L^1^ and (b) thin film of MFM-186 in CH_2_Cl_2_ containing [*^n^*Bu_4_N][BF_4_] (0.4 M). The background noise observed here is relatively high as a result of low quantities of MFM-186 being deposited and the discontinuous nature of the MOF film. Potentials are reported *vs.* Fc^+^/Fc. Kohn–Sham frontier orbital (HOMO) of (c) Et_4_L^1^ and (d) model system for MFM-186. Colour code: C: dark grey, H: light grey, Cu: blue, O: red, HOMO: green and yellow.

To understand these redox processes in detail we decided to monitor each process using UV-Vis absorption spectroscopy (Fig. 4a and b). The spectral changes in the MOF films on ITO-coated glass were compared with those observed for the solutions of the ester derivatives of the corresponding ligand in the same electrolyte solution. During this study, the working electrode was fixed at a constant overpotential to achieve the desired redox reaction, while UV-Vis absorption spectra were collected at regular time intervals. The spectral changes observed due to oxidation of Et_4_L^1^ at 1.14 V are characterised by the disappearance of π–π* transitions of the neutral species around *λ*_max_ ≈ 350–410 nm concomitant with the appearance of new absorption bands around *λ*_max_ ≈ 550–700 nm assigned to π–π* transitions of the oxidized species. This suggests the formation of a ligand-centred radical cation species.^41^ The presence of an isosbestic point at *λ*_iso_ ≈ 410 nm indicates that the electrochemical oxidation of Et_4_L^1^ is reversible and occurs without significant chemical degradation or any long-lived intermediates. Furthermore, the chemical stability of the oxidised species under these conditions was confirmed by the regeneration of the spectral profile of the starting material Et_4_L^1^ upon application of potential sufficiently negative to reduce the oxidised species. The oxidation of films of MFM-186 displays similar behaviour to those above, albeit that the bands are slightly red shifted, consistent with the observed oxidation of the MOF being ligand-centred.

The first oxidation of a molecular system is often considered to be directly related to the energy of the highest occupied molecular orbital (HOMO) of that system. Therefore, to better understand the nature of the oxidation process of Et_4_L^1^ and to support the above assignment that the oxidation in the MOF is ligand-centred we have carried out density-functional-theory (DFT) calculations to identify the HOMO. The calculated HOMO for Et_4_L^1^ is almost entirely centred on the anthracene moiety (Fig. 4c), a result that is unsurprising given the similarity of the UV-Vis absorption spectrum to that of many other anthracene-bearing molecules.^41^ To compare the orbital character of the valance band of MFM-186 with the HOMO for Et_4_L^1^, a simplified model of MFM-186 was considered. The model consisted of four Cu(ii) paddlewheel clusters connected *via* one ligand and truncated by benzoate ions. The DFT calculations for this model show that the HOMO within MFM-186 is anthracene-based (Fig. 4d) supporting the oxidation of MFM-186 being ligand-centred.

### Bulk electrochemical analysis of MFM-180

**Fig. 5:**
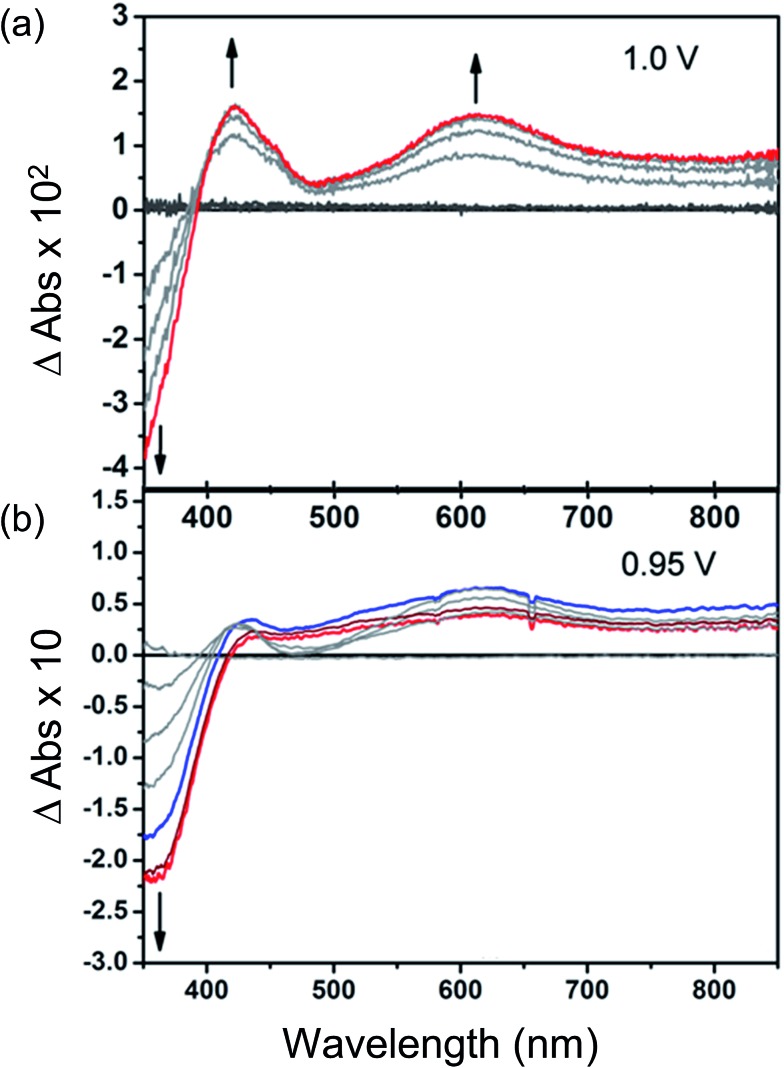
Time-dependent difference spectra of electrochemically oxidised (a) Et_8_L^2^ and (b) thin film of MFM-180. Potentials are reported *vs.* Fc^+^/Fc. The blue trace in (b) indicates the point when concentration of the oxidised species [MFM-180]˙^+^ is highest (∼8 min), while the red trace indicates the final concentration after ∼15 min.

Changes in the UV/Vis spectrum of Et_8_L^2^ upon oxidation (at 1.0 V) are characterised by the disappearance of π–π* transitions of the neutral species around *λ*_max_ ≈ 350–410 nm (Fig. 5). The disappearance of this band coincides with the appearance of a new absorption feature around *λ*_max_ ≈ 600 nm indicating the formation of a radical cation species.^41^ However, in this case, no isosbestic points were observed during the progress of the oxidation. Furthermore, the starting material was not regenerated completely upon reduction of the oxidised species indicating instability of the electrogenerated radical ion.

**Fig. 6:**
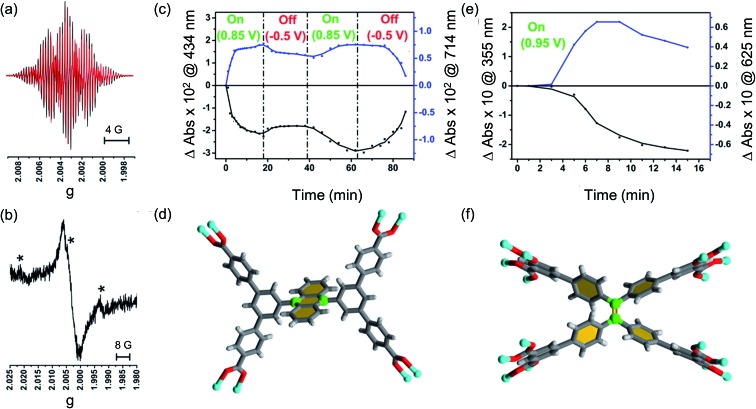
EPR spectra of (a) oxidised Et_4_L^1^ (black trace: experimental, red trace: simulated) and (b) oxidized film of MFM-186. The mark ‘*’ denotes a possible impurity from NO from the reduction of NOBF_4_. The time-dependent changes in UV-Vis absorbance for (c) thin film of MFM-186 and (e) thin film of MFM-180 recorded as a response towards electrochemical oxidation at ambient temperature at specific potentials. Black traces in both cases indicates, π–π* transitions of neutral species whereas blue traces show changes in π–π* transitions of oxidized species. Also shown coordinating environment of the ligand (d) [Et_4_L^1^]^4–^ and (f) [Et_8_L^2^]^8–^. Two carbon atoms for a possible site of the nucleophilic attack have been highlighted in green. Colour code: C: dark grey, H: light grey, Cu: blue, O: red. Note; in case of (d) two carbon atoms for nucleophilic attack are more sterically crowded.

As expected, the MFM-180 film shows similar spectral behaviour to Et_8_L^2^ upon oxidation (0.95 V). Oxidation results in the disappearance of the π–π* transition band at 350–410 nm and the concomitant formation of a band at ∼625 nm in the UV-Vis absorption spectrum attributed to the generation of a radical cation species. However, the oxidised MFM-180 film during these experiments degrades with loss of the band at 625 nm. This is illustrated by a plot of the changes in absorbance at 355 nm and 625 nm against time (Fig. 6e) suggesting that the radical cation [MFM-180]˙^+^ decomposes, possibly *via* a non-redox chemical transformation.^40^ This is in contrast to the spectroelectrochemical investigation of [MFM-186]˙^+^, generated *via* electrochemical oxidation of MFM-186, which shows the radical cation to be stable. The concentration of this radical species can be controlled by switching the bias on and off (Fig. 6c).

### EPR analysis of [MFM-186]˙^+^

To better understand the nature of the SOMO of oxidized MFM-186, we recorded EPR spectra of oxidized Et_4_L^1^ (*i.e.*, [Et_4_L^1^]˙^+^) as a fluid solution and MFM-186 (*i.e.*, [MFM-186]˙^+^) powder, each at ambient temperature. The radical cation of Et_4_L^1^ was generated electrochemically in CH_2_Cl_2_ containing [*^n^*Bu_4_N][BF_4_] (0.4 M), whilst the oxidation of the MOF was achieved chemically using NOBF_4_ in CH_2_Cl_2_ (ref. 42) as the oxidant. The experimental EPR spectrum of oxidized Et_4_L^1^ can be reasonably reproduced by simulation using the hyperfine couplings of three sets of equivalent protons (*g*_iso_ = 2.0027, *a*_4H_ = 2.553 × 10^–4^ cm^–1^, *a*_4H_ = 1.161 × 10^–4^ cm^–1^, *a*_6H_ = 0.382 × 10^–4^ cm^–1^). This suggests that the SOMO for [Et_4_L^1^]˙^+^ is localised mainly on anthracene core but some electron density is delocalised onto the two adjoining 3,5-substituted phenyl rings (Fig. 6, S10† and related discussion). Although we failed to resolve hyperfine coupling in oxidized MFM-186 powder, *g*_iso_ values are consistent with an organic based radical and the spectral width is similar to that of [Et_4_L^1^]˙^+^ suggesting a common location for the SOMO.

In order to explain the difference in stability between [MFM-186]˙^+^ and [MFM-180]˙^+^ we note that in the crystal structure of MFM-186 the 9- and 10-positions of the anthracene (the reactive part of the anthracene core) are sterically crowded (Fig. 6d) which may make the radical cation [MFM-186]˙^+^ kinetically more stable to nucleophilic attack. The redox active ‘tetraphenylethylene’ core in MFM-180 lacks this protection which makes [MFM-180]˙^+^ more susceptible to attack by any nucleophile present. We, therefore, suggest that presence of any water or other nucleophilic guest trapped in the MOF framework scavenges the radical in [MFM-180]˙^+^. Although in principle a ligand–ligand dimerization reaction pathway is also possible, we suggest that such a pathway may not be favourable due to the long ligand–ligand distance in the crystal structure (Fig. S14†).^43^

Unfortunately, when attempting to study the reduction properties of MFM-186 and MFM-180 both samples underwent film delamination. This is not entirely unexpected since reduction processes will be based either at the Cu(ii) paddlewheel centre or at the carboxylates, and this will drive significant changes in the coordination geometry and ligand binding leading to destabilisation of the overall framework structure and delamination of the film.

## Conclusions

A method of generating surface bound MOFs to study their electrochemical properties has been developed and has been described for two samples. Both MFM-180 and MFM-186 can be oxidized to generate radical cationic species, [MFM-180]˙^+^ and [MFM-186]˙^+^, respectively. Though [MFM-180]˙^+^ is unstable, [MFM-186]˙^+^ is stable to redox cycling. The methodology outlined in this work allows for the design of new materials for electrocatalysis^44^ and provides a framework by which such materials may be studied using *in situ* spectroscopic methods. The success of this spectro-electrochemical approach relies on our technique of covalently attaching stable MOF films on conducting working electrode surfaces *via* functionalization of the surface with a “chemical glue” onto which can be attached a thin film of MOF. Covalent attachment allows the MOF framework to be securely attached on to an electrode surface. It has been shown previously that electrons are transferred between electrode and substrate through immobilized conjugated “molecular bridges” more rapidly than in the absence of such bridges.^40^,^45^ In the present case, the immobilized contact between substrate (MOF) and the electrode is likely to favour electron transfer between them. This also circumvents reliance upon deposition of material onto a surface by mechanical means. The covalently-bound, surface-modifying carboxylate species is likely to affect the orientation and growth of MOF crystallites on a surface, an area in which our investigations are ongoing. Also, this method does not involve corrosive reagents, for example, inorganic acids often needed for MOF synthesis, which may harm fragile and costly conducting surfaces. This new approach can potentially open up a new pathway to fabricate MOF-modified electrodes or semiconductor based devices.

## Conflicts of interest

There are no conflicts to declare.

## Supplementary Material

Supplementary information
