# Supplementary material for: Characterisation of redox states of metal–organic frameworks by growth on modified thin-film electrodes
Source: Chem Sci. 2018 Jun 4;9(31):6572–9. doi: 10.1039/c8sc00803e (PMC6115678; doi:10.1039/c8sc00803e)
Supplement: Supplementary file 1 [file SC-009-C8SC00803E-s001.pdf]

Electronic Supplementary Material (ESI) for Chemical Science.
This journal is © The Royal Society of Chemistry 2018

Supporting Information

Characterisation of Redox States of Metal-Organic Frameworks by Growth on Modified Thin-film Electrodes

Tamoghna Mitra,[a,b] Florian Moreau,[a,c] Adam Nevin,[a,c] Carlo U. Perotto,[a] Alex Summerfield,[d] E. Stephen Davies,[a] Elizabeth A. Gibson,[a,e] Timothy L. Easun,[a,f,]* and Martin Schröder[a,b]*

a. School of Chemistry, The University of Nottingham, University Park Nottingham, NG7 2RD, UK

b. Department of Chemistry, The University of Liverpool, Crown Street, Liverpool, L69 7ZD, UK

c. School of Chemistry, The University of Manchester, Oxford Road, Manchester M13 9PL, UK

d. School of Physics and Astronomy, The University of Nottingham, University Park Nottingham, NG7 2RD, UK

e. School of Chemistry, Bedson Building, Newcastle University, Newcastle upon Tyne NE1 7RU, UK

f. School of Chemistry, Cardiff University, Park Place, Cardiff, CF10 3AT, UK

Experimental Section

Instrumentation. Electrochemical measurements were made using an Eco Chemie Autolab PGSTAT20 potentiostat. All solutions were purged with a stream of Ar prior to use. Cyclic voltammetry was performed using a three-electrode system, with a Pt wire secondary electrode and a saturated calomel reference electrode. For solution based cyclic voltammetry a glassy carbon working electrode was used and before each measurement the electrode was cleaned using a polishing pad. All potentials are referenced to the Fc^+/Fc couple used as an internal standard. Cyclic voltammograms were recorded for solutions of compounds (*ca.* 1 mM) in the presence of $[^nNBu_4][BF_4]$ (0.4 M) as supporting electrolyte. Coulometric measurements were performed in an H-cell at 273 K in CH_2Cl_2 containing $[^nNBu_4][BF_4]$ (0.4 M); the cell consisted of a Pt/Rh gauze basket working electrode separated by a glass frit from a Pt/Rh gauze secondary electrode. A saturated calomel reference electrode was bridged to the test solution through a Vycor frit oriented at the centre of the working electrode, and the solution was stirred rapidly during electrolysis using a magnetic stirrer bar.

UV-visible spectroelectrochemical measurements on Et_4L^1 and Et_8L^2 were carried out using an optically transparent electrode mounted in a modified quartz cuvette with an optical path length of 0.5 mm. A three-electrode configuration consisting a Pt/Rh gauze working electrode, a Pt wire secondary electrode (in a fritted PTFE sleeve) and a saturated calomel electrode, chemically isolated from the test solution *via* a bridge containing electrolyte solution and terminated in a porous frit, was used in the cell. The potential at the working electrode was controlled by a Sycopel Scientific Ltd DD10M potentiostat. The UV-visible spectra were recorded on a Perkin Elmer Lambda 16 spectrophotometer. The spectrometer cavity was purged with N_2 and temperature control at the sample was achieved by flowing cooled N_2 across the surface of the cell. UV-visible spectroelectrochemical experiments on thin films of MOFs were carried out by using an Ocean Optics Jaz spectrometer equipped with tungsten and

deuterium light sources. X-band EPR spectra were recorded on a Bruker EMX spectrometer. The simulations of the EPR spectra were performed using the Bruker WINEPR SimFonia package. Powder X-ray diffraction patterns were collected on a Panalytical diffractometer using Cu-Kα radiation (λ = 1.5418 Å) in reflection mode.

AFM measurements were recorded on films grown on the ITO surface by loading the MOF-modified-substrate into an Asylum Research Cypher-S AFM. Repulsive-mode amplitude-modulated AFM images were obtained using Olympus AC240-TS AFM cantilevers (Asylum research). AFM images were processed using the Gwyddion software package.

Unless otherwise stated, reagents were used as received from the suppliers (Sigma-Aldrich, Acros Organic and Fluka) and all reactions, manipulations and transfers were performed under an inert atmosphere of Ar using standard Schlenk techniques. For electrochemical analysis, solvents were dried and degassed following standard procedures and stored under Ar in Young's ampoules over molecular sieves (pore size 4 Å). High-purity Ar was obtained from BOC gases and used without any further purification. Indium doped tin oxide glass (150 nm coating, 12 ohms per square) was obtained from Visiontek Glass and cleaned by washing and sonicating in acetonitrile. Conducting carbon paper was obtained from SGL and was cleaned in a similar way before use.

H_8L^2, Et_8L^2 (H_8L^2 = 4',4''',4''''',4'''''''-(ethene-1,1,2,2-tetrayl)tetrakis(([1,1'-biphenyl]-3,5-dicarboxylic acid) and Et_8L^2 = octaethyl 4',4''',4''''',4'''''''-(ethene-1,1,2,2-tetrayl)tetrakis([1,1'-biphenyl]-3,5-dicarboxylate)) were synthesized as reported previously.[S1] H_4L^1 and Et_4L^1 (H_4L^1 = 5', 5''''-(anthracene-9,10-diyl)bis(([1,1':3',1''-terphenyl]-4,4''-dicarboxylic acid)), Et_4L^1 = tetraethyl 5',5''''-(anthracene-9,10-diyl)bis([1,1':3',1''-terphenyl]-4,4''-dicarboxylate) were synthesized as follows.

Scheme S1: Synthesis of Et_4L^1 and H_4L^1

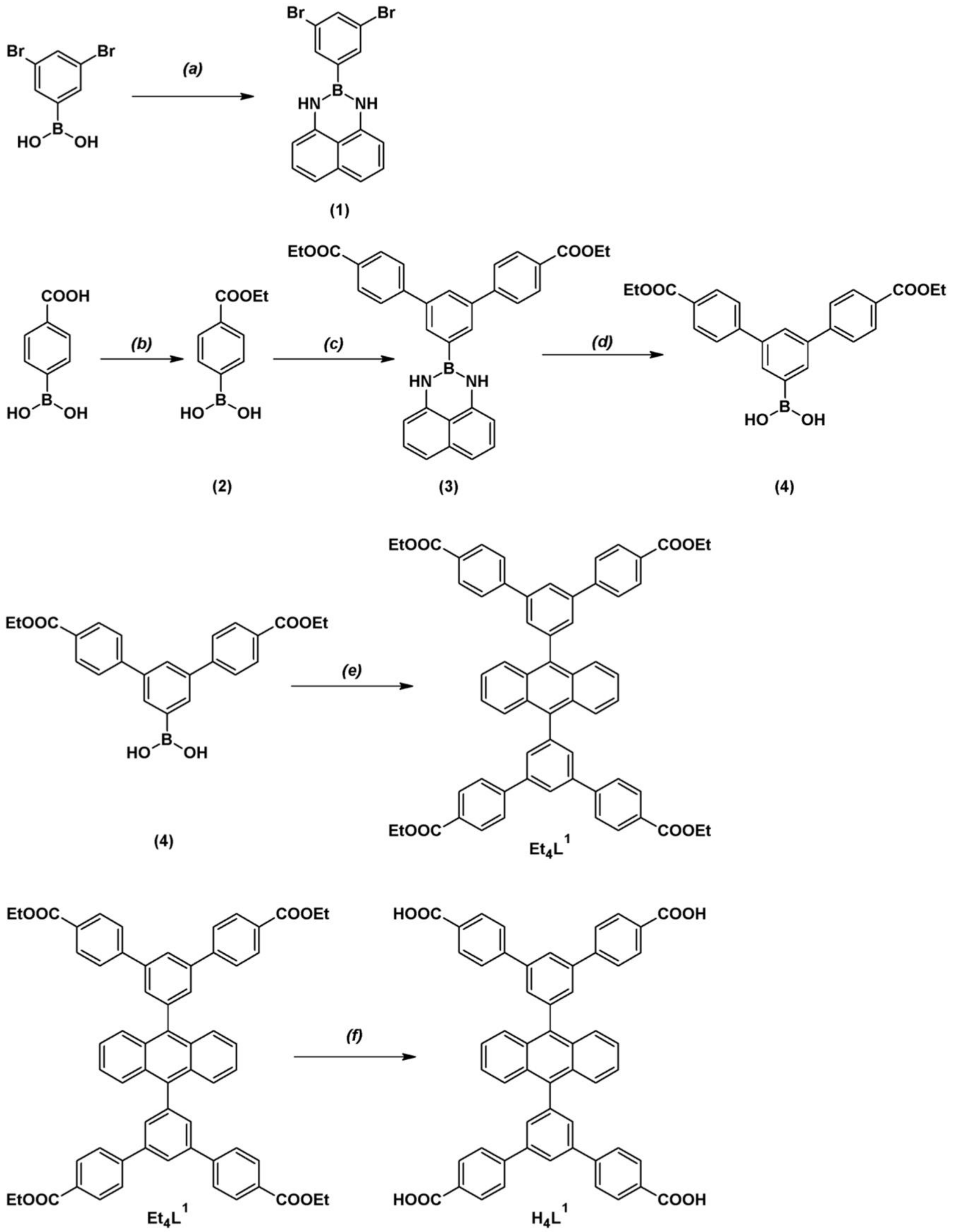

(a) : 1,8-diaminonapthalene, toluene, 100 °C 1 h
(b) : Ethanol, Conc. Sulphuric acid, reflux 19 h
(c) : (i) (1), Potassium Carbonate, Toluene/water 60 °C, Ar
(ii) Tri-^{t}butyl phosphine, tris(dibenzylideneacetone)dipalladium(0), 80 °C 90 min, Ar
(d) : THF, 2M sulphuric acid, reflux 4 h
(e) : (i) 9,10 dibromo anthracene, Potassium Carbonate, Toluene/water 60 °C, Ar
(ii) Tri-tbutyl phosphine, tris(dibenzylideneacetone)dipalladium(0), 80 °C 150 min, Ar
(f) : (i) THF/EtOH, 2M NaOH(aq), reflux, 23 h; (ii) HCl(aq)

Synthesis of Et_4L^1 and H_4L^1

Synthesis of 2-(3,5-dibromophenyl)-2,3-dihydro-1H-naphtho[1,8-de][1,3,2]diazaborinine, 1.

To a stirred solution of (3,5-dibromophenyl) boronic acid (10.0 g, 36 mmol) in toluene (170 mL) was added 1,8-diaminonapthalene (6.6 g, 42 mmol). The solution was heated to 100 °C for 1 h and the solvent removed under reduced pressure to give the crude product as a brown solid. The solid was dissolved in a minimum volume of boiling CH_2Cl_2, and the crude product precipitated by addition of petroleum ether (b.p 60-80 °C). The suspension was allowed to cool to room temperature, and the solid was separated by filtration and dried (80 °C) to obtain pure product as a bright yellow solid (11.3 g, 78%). Spectroscopic analysis and purity of the compound were in accordance to those found in literature.[S2]

Synthesis of 4-ethoxycarbonylphenyl boronic acid, 2

To a stirred solution of 4-carboxyphenylboronic acid (15.0 g, 90 mmol) in EtOH (375 mL) was added concentrated H_2SO_4 (4.5 mL). The solution was heated at reflux for 19 h and the solution concentrated under reduced pressure until a precipitate formed. An excess of water was added to the suspension, which was collected by filtration. The solid product was washed with water until the filtrate was pH 7, and then dried (80 °C) to give the product as a fine white powder (15.4 g, 88%).

Synthesis of 5'-(1H-naphtho[1,8-de][1,3,2]diazaborinin-2(3H)-yl)-[1,1':3',1''-terphenyl]-4,4''-dicarboxylate diethyl ester, 3

To a stirring, degassed suspension of **1** (6.0 g, 15 mmol), **2** (8.13 g, 41 mmol) and K_2CO_3 (4.34 g, 44 mmol) in toluene (400 mL) and water (100 mL) at 60 °C was added tri-tert-butyl phosphine (1M in toluene, 2.4 mL, 2.4 mmol) and tris(dibenzylideneacetone)dipalladium(0) (1.34 g, 1.5 mmol). The reaction mixture was heated to 80 °C for 1.5 hours, and the resulting

suspension filtered while hot. The solid was extracted with CH_2Cl_2 (200 mL) and the organic phase washed with water (2 x 200 mL), dried over $MgSO_4$ and filtered. The solvent was removed under reduced pressure and the resulting black oil was dissolved in a minimum volume of CH_2Cl_2 and the solution passed through a silica plug using ethyl acetate as eluent. The filtrate was evaporated to dryness under reduced pressure to give a dark brown solid. The solid was dissolved in a minimum volume of boiling CH_2Cl_2, and precipitated by addition of petroleum ether (b.p. 60-80 ^{o}C). The product was separated by filtration and dried (80 °C) to obtain the pure product as a yellow/orange solid (5.98 g, 74%). (Found: [M], 540.13. $C_{34}H_{29}O_4N_2B$ requires 540.22); ^{1}H NMR (300MHz, $CDCl_3$): δ = 1.46 (t, $^{3}J_{H,H}$ 7.1 Hz, 6H, CH_3), 4.45 (q, $^{3}J_{H,H}$ 7.1 Hz, 4H, CH_2), 6.15 (s, 2H, NH), 6.49 (d $^{3}J_{H,H}$ 7.1 Hz, 2H, Ar), 7.10-7.22 (m, 4H, Ar), 7.78 (dt, $^{3}J_{H,H}$ 8.5 Hz, $^{3}J_{H,H}$ 1.9 Hz, 4H, Ar), 7.90 (d, $^{3}J_{H,H}$ 1.8 Hz, 2H, Ar), 7.94 (t, $^{3}J_{H,H}$ 1.8 Hz, 1H, Ar), 8.20 (dt, $^{3}J_{H,H}$ 8.5 Hz, $^{3}J_{H,H}$ 1.9 Hz, 4H, Ar) ppm. ^{13}C NMR (100 MHz, $CDCl_3$): δ = 14.42, 61.06, 106.19, 118.22, 127.30, 127.63, 127.69, 128.19, 129.75, 130.14, 130.24, 140.82, 140.89, 145.17, 166.44 ppm.

Synthesis of 4,4''-bis(ethoxycarbonyl)-[1,1':3',1''-terphenyl]-5'-yl)boronic acid, 4

To a stirred solution of **3** (6.35 g, 11.8 mmol) in tetrahydrofuran (360 mL) was added H_2SO_4 (2M, 73 mL) and the solution heated under reflux for 4 h. The reaction mixture formed a suspension over this time, and this was filtered while hot to remove the solid impurities. The filtrate was reduced under reduced pressure until a precipitate formed. This suspension was re-solubilised by heating to 70 °C, and an excess of water was added to precipitate a solid. The mixture was filtered and the solid collected and washed with a large volume of water until the filtrate was at pH = 7. The product was dried (80 °C) to give an off white solid (4.44 g, 97%). (Found: [M - H], 417.1585. $C_{24}H_{22}O_6B^{-}$ requires 417.1509); ^{1}H NMR (300MHz, $CDCl_3$): δ = 1.36 (t, $^{3}J_{H,H}$ 7.1 Hz, 6H, CH_3), 4.36 (q, $^{3}J_{H,H}$ 7.1 Hz, 4H, CH_2), 7.96 (d $^{3}J_{H,H}$ 8.3 Hz, 4H, Ar), 8.08 (d, $^{3}J_{H,H}$ 8.3 Hz, 5H, Ar), 8.21 (d, $^{3}J_{H,H}$ 1.8 Hz, 2H, Ar) ppm. ^{13}C NMR

(100 MHz, CDCl3): δ = 14.67, 61.33, 86.17, 92.38, 127.62, 128.43, 129.23, 130.23, 139.24, 145.25, 166.08 ppm.

Synthesis of Et_4L^1 (tetraethyl 5',5''''-(anthracene-9,10-diyl)bis([1,1':3',1''-terphenyl]-4,4''-dicarboxylate)

To a stirring, degassed solution of 9,10-dibromoanthracene (474 mg, 1.41 mmol), **4** (1.65 g, 4.2 mmol) and K_2CO_3 (1.09 g, 11 mmol) in toluene (133 mL) and water (34 mL) at 60 °C was added tri-tert-butyl phosphine (1M in toluene, 0.65 mL, 0.65 mmol) and tris(dibenzylideneacetone)dipalladium(0) (258 mg, 0.28 mmol). This suspension was heated at 80 °C for 2.5 hours. The workup procedure was the same as for **3**. The product was a yellow solid (1.06 g, 82%). (Found: [M], 922.21. $C_{62}H_{50}O_8$ requires 922.35); ^{1}H NMR (300MHz, $CDCl_3$): δ = 1.45 (t, $^3J_{H,H}$ 7.2 Hz, 12H, CH_3), 4.44 (q, $^3J_{H,H}$ 7.2 Hz, 8H, CH_2), 7.40-7.47 (m, 4H, Ar), 7.80-7.90 (m, 16H, Ar) 8.10 (t, $^3J_{H,H}$ 1.7 Hz, 2H, Ar), 8.18 (dt, $^3J_{H,H}$ 8.5 Hz, $^3J_{H,H}$ 1.9 Hz, 8H, Ar) ppm. ^{13}C NMR (100 MHz, CDCl3): δ = 14.47, 61.13, 86.17, 108.20, 125.62, 127.22, 130.23, 131.43, 139.44, 141.01, 141.04, 149.25, 156.86, 171.28, 171.48 ppm.

Synthesis of H_4L^1 5',5''''-(anthracene-9,10-diyl)bis([1,1':3',1''-terphenyl]-4,4''-dicarboxylic acid).

To a stirred solution of Et_4L^1 (1.00 g, 1.1 mmol) in tetrahydrofuran (140 mL) and EtOH (140 mL) was added NaOH (2M, 140 mL), and the reaction mixture heated at 100 °C for 23 h. The cooled solution was filtered, and the filtrate was observed to have two phases. The aqueous phase was separated, acidified with HCl (pH ~1), and the resulting suspension was filtered and the solid collected and washed with water until the filtrate was at pH = 7. The product was then dried (80 °C) to give a brown solid (874 mg, 99%). (Found: [M], 810.06. $C_{54}H_{34}O_8$ requires 810.23); ^{1}H NMR (300MHz, $CDCl_3$): δ = 7.43-7.55 (m, 4H, Ar), 7.73-7.83 (m, 4H, Ar), 7.90 (s, 4H, Ar), 8.06 (s, 16H, Ar), 8.35 (s, 2H, Ar), 13.02 (s, 4H, COOH). ^{13}C NMR

(100 MHz, CDCl3): δ = 108.00, 111.40, 123.42, 123.82, 127.82, 128.23, 129.83, 130.43, 139.04, 140.84, 144.05, 156.66 ppm. CHN – Expected: C, 79.99; H, 4.23; O, 15.79, Found: C, 75.8; H, 4.2.

Synthesis of MFM-186 as a solid crystalline powder.

H_4L^1 (10 mg, 0.013mmol) and $Cu(NO_3)_2.2.5H_2O$ (26 mg, 0.11 mmol) were dissolved in DMF (3 mL) and the reaction mixture acidified with aq. HCl (3 drops, 8M). The reaction mixture was heated at 80 ^oC for 16 h in a sealed glass vial (9 mL). After 16 h greenish blue crystals of MFM-186 were collected by filtration, washed with DMF and dried under vacuum. Yield 60%.

Functionalisation of conducting surface.

Indium tin oxide (ITO) coated glasses and carbon paper was treated before each experiment by ultrasonic cleaning in ultrapure H_2O for 15 mins, followed by ultrasonication for 30 min in CH_3CN prior to modification. The diazonium reduction experiment was carried out with 1 mM diazonium salt in a solution of 0.1 M [$^nNBu_4$][ClO_4] in CH_3CN under an inert atmosphere. The modified electrodes were washed with CH_3CN and then ultrasonicated in CH_3CN for 30 min and stored in dry CH_3CN under an Ar atmosphere. The barrier properties of the unmodified and the modified electrodes were evaluated in 1 mM ferrocene solution with 0.1M [$^nNBu_4$][ClO_4] in dry CH_3CN.

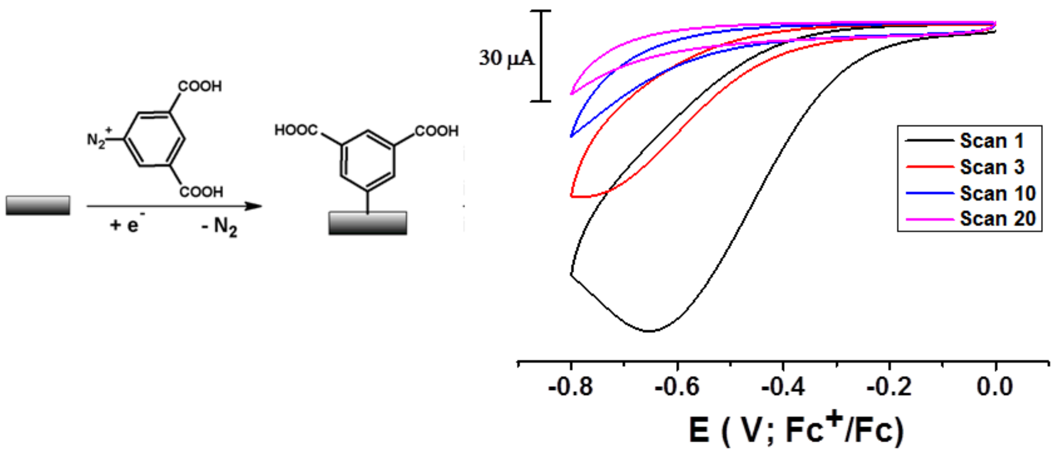

Figure S1: Cyclic voltammograms of an ITO-glass electrode in 1mM diazonium salt in a solution of 0.1 M [^{n}NBu$_4$][ClO$_4$] in CH_3CN. Scan rate = 100 mV/s.

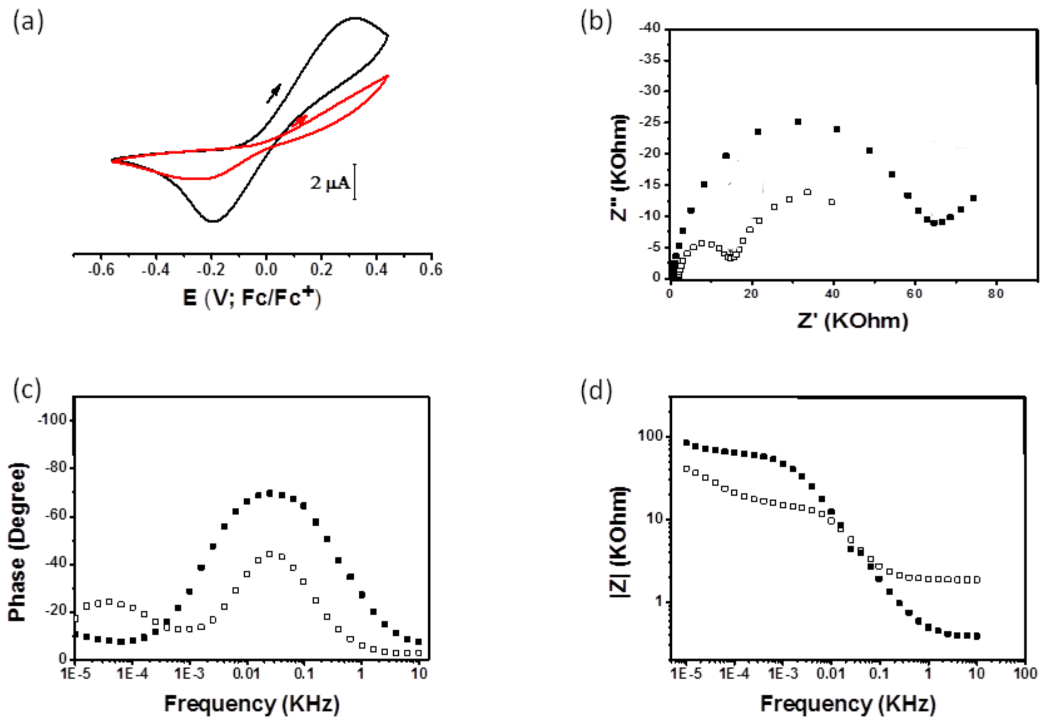

Figure S2: Blocking behaviour of isophthalic acid coated ITO-glass electrode. (a) Cyclic voltammograms of ferrocene solution at a scan rate of 10 mV/s – before (black trace) and after (red trace) coating with isophthalic acid, (b) Nyquist plot and (c-d) Bode plots of ITO glass electrode (open symbol) and isophthalic acid coated electrode (close symbol).

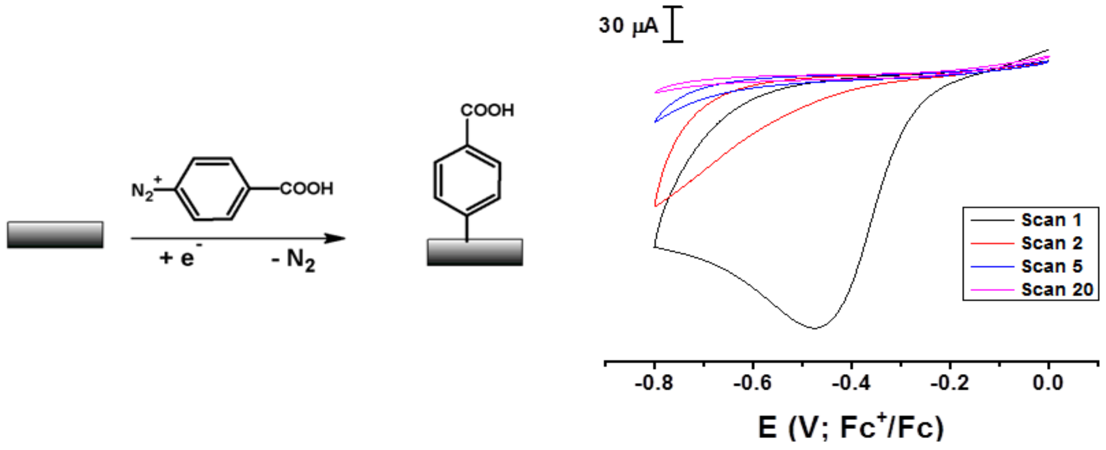

Figure S3: Cyclic voltammograms of an ITO-glass electrode in 1 mM diazonium salt/0.1 M [^{n}NBu$_4$][ClO$_4$]/CH$_3$CN solution: Scan rate = 100 mV/s.

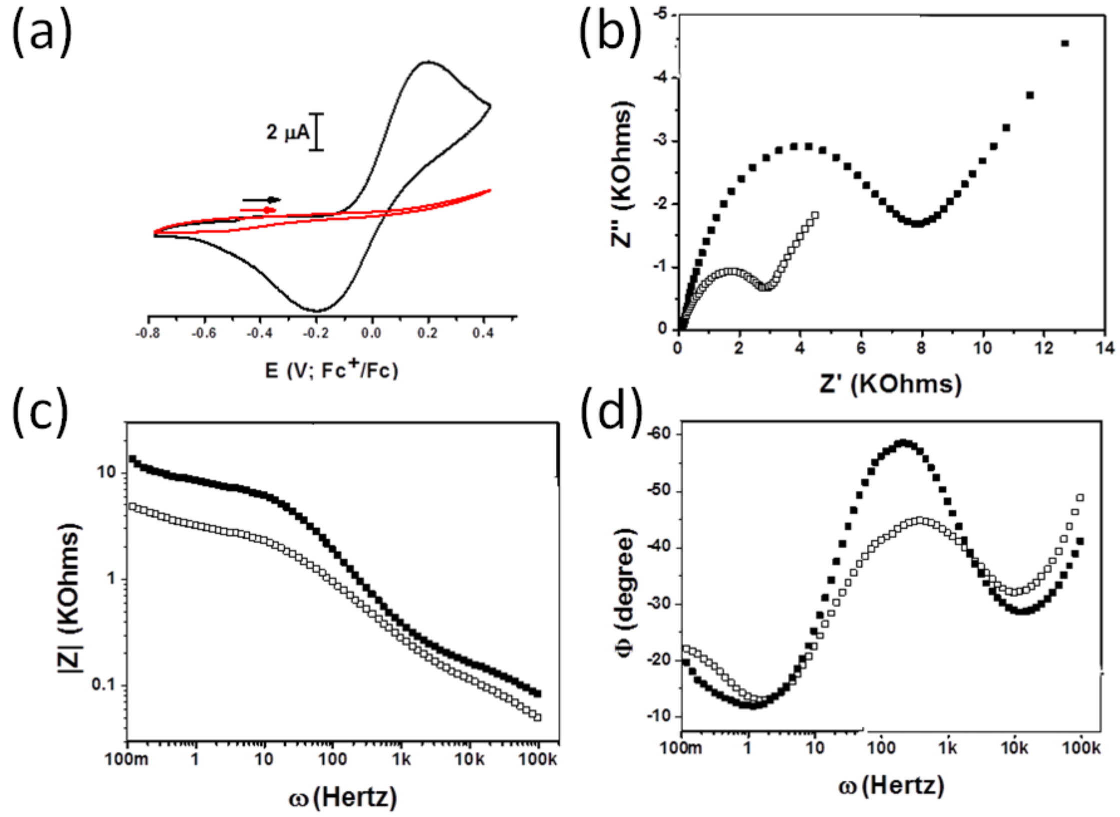

Figure S4: Blocking behaviour of benzoic acid coated ITO-glass electrode. (a) Cyclic voltammograms of ferrocene solution at a scan rate of 10 mV/s – before (black trace) and after (red trace) coating with benzoic acid, (b) Nyquist plot and (c-d) Bode plots of ITO glass electrode (open symbol) and isophthalic acid coated electrode (close symbol).

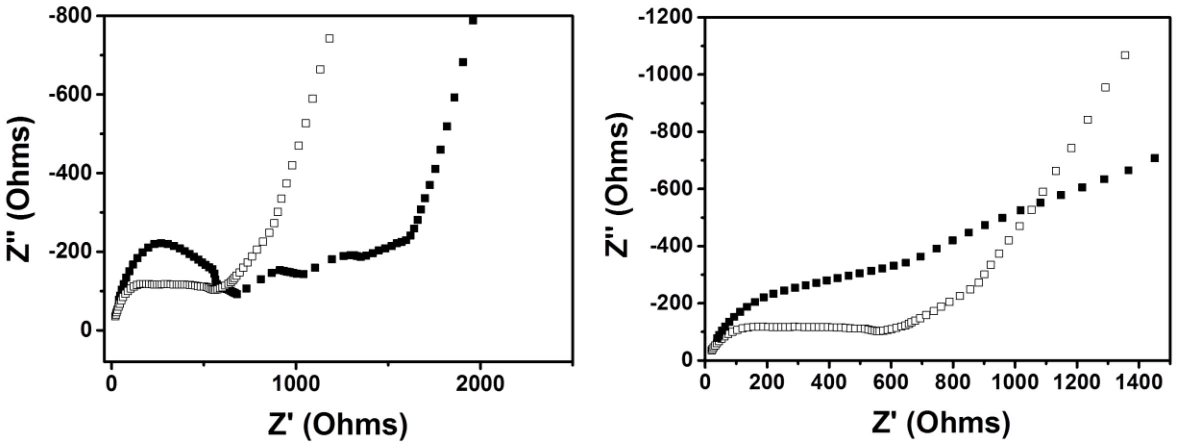

Figure S5: Blocking behaviour of Aryl monolayer coated carbon paper electrode. (~ 1 mm x 1mm): Nyquist plot of (left) isophthalic acid coated C-paper electrode and (right) benzoic acid coated C-paper electrode before and after coating.

Modification of conducting surface with MOF coatings: Separate solutions of ligand (0.2 mM) and $[Cu(OAc)_2].H_2O$ (0.4 mM for MFM-186 film and 0.8 mM for MFM-180) in DMF were freshly prepared for each experiment. Typical experiments were carried out using an in-house-built dip coating system. The conducting surface was immersed sequentially first in $[Cu(OAc)_2].H_2O$ solution and then into the ligand solution for 1 min each. The metal ion solution and ligand solution were not stirred during this process. Between each dipping, the surface was washed by immersion in stirring DMF for 2 min. This whole sequence is considered as one complete cycle. For characterization by PXRD a film with 30 such cycles of dipping was synthesized, while for all electrochemical studies films with 10 such cycles were synthesized. The AFM studies were conducted on films grown by 10 and 20 cycles of sequential dipping.

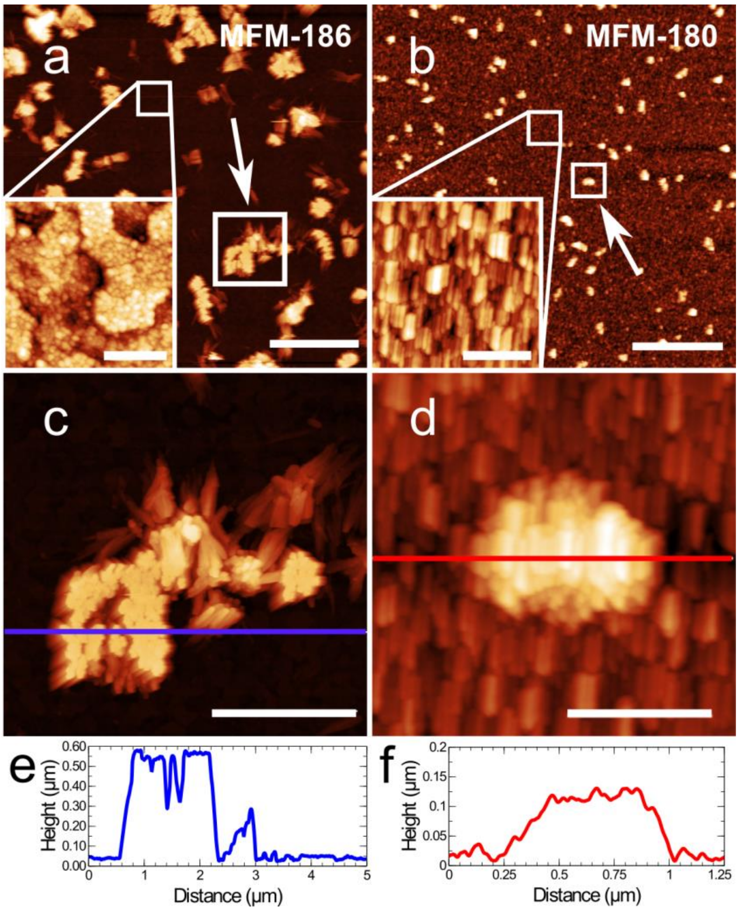

Figure S6: AFM images of (a,c) MFM-186 and (b,d) MFM-180 grown on functionalised ITO substrate after 10 cycles of deposition. Extra crystallites also grow on the surface, apparently not connected to the cyclic deposition. a) AFM image of MFM-186 on ITO; the arrow indicates the region in image c. Scale bar 5 µm. (inset) Image of the region between the agglomerated crystallite deposits of MFM-186. Scale bar 300 nm. b) AFM image of MFM-180 on ITO, the arrow indicates the region in image d. Scale bar 5 µm. (inset) Image of the region between the agglomerated crystallite deposits of MFM-180, which is also covered by closely packed crystallites of MFM-180, leading to a continuous coverage of MFM-180 on the surface. Scale bar 400 nm. c) High resolution image of the MOF deposit indicated by the arrow in image a. Scale bar 2 µm. d) High resolution image of the region indicated by the arrow in image b. Scale bar 400 nm. e) Height profile along the region marked by the blue line in image c. f) Height profile along the region marked by the red line in image d.

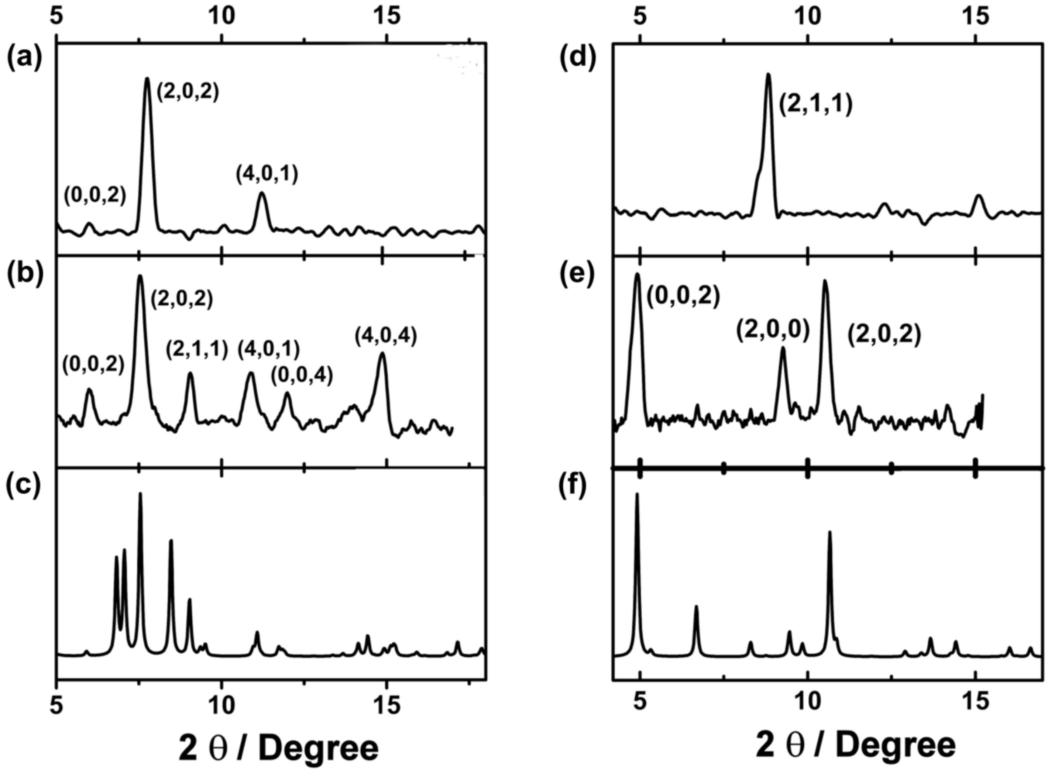

Figure S7: PXRD of **MFM-186** films on (a) ITO electrode, on (b) carbon paper, (c) simulated from single crystal structure, and **MFM-180** films (d) on ITO electrode, on (e) carbon paper, (f) simulated from single crystal structure.

Cyclic Voltammetry (CV) and Bulk Electrolysis of MOF Films.

For all electrochemical studies, MOF films were synthesized from 10 dipping cycles. For cyclic voltammetry, the MOF films grown on carbon paper (1mm x 1mm) were used as working electrodes due to the superior conductivity of carbon paper over an ITO-coated glass. Conversely, the ITO-based films were used as working electrodes for spectroelectrochemical studies to exploit the transparent nature of the ITO-coated glass. Prior to electrochemical experiments the MOF films were immersed in CH_2Cl_2 for 3-4 days, with solvent replaced every 24h. The films were then dried in air and re-immersed in the electrolyte solution of 0.4 M of $[^nBu_4N][BF_4]$ in CH_2Cl_2 for 2-3 days to allow CH_2Cl_2 and electrolyte to fully exchange with

the other guests such as DMF trapped in the MOF pores. This acts as an "electrochemical conditioning" period.

We found that it is necessary to use thinner films (10 cycles of dipping) in order to record the voltammetric response. This can be explained by considering ion transfer through MOF pores. The distance an ion can travel/diffuse during an electrochemical process is usually proportional to $(Dt)^{0.5}$ (where D = diffusion coefficient, t = time).[S3] Therefore it is necessary for films to be thinner than the diffusion layer so that electrolyte can diffuse readily through the pores of the MOF to the electrode surface.

CV Scan Rate (ν) Dependence of Peak Currents (i_p) and Separation (ΔE_p) in MFM-186. CV measurements of on MFM-186 films on carbon paper were carried out at a series of scan rates (ν = 2, 3, 4, 5, 6, 8, 10 mV/s). The resulting data are plotted in Figure S8. For diffusion limited processes in solution-based measurements, i_p is directly proportional to $\nu^{1/2}$ while ΔEp should be 0.059/n V at 25 °C. However, for surface-confined processes not subject to diffusion limitation, i_p should be directly proportional to ν and $\Delta E_p = 0$ V. The ΔE_p *vs* scan rate plot (Figure S8) shows that ΔE_p for MFM-186 is not zero, though values are far less than 59mV. To understand the dependence of i_p over scan rate a Ln(i_p) *vs* Ln(scan rate) plot can be used.

$i_p \propto \nu^n$ where ν = scan rate; $1 \geq n \geq 0.5$

Hence,

$\mathrm{Ln}(i_p) = n\mathrm{Ln}(\nu) + \text{constant}.$ $[\mathrm{Ln}(x) = \log_e(x)]$

Therefore, the slope for the data plotted as Ln(i_p) *vs* Ln(ν) can indicate the order of the dependence with respect to scan rate. For a diffusion limited process, the slope of this plot should approach 0.5, while for a surface confined process the slope should be 1.

For MFM-186 film the slope tends to be closer to 1 for slow scan rates although this deviates from ideal behaviour at the higher scan rate. Such behaviour is not expected from a surface confined species. This phenomenon has been observed in related literature and is explained by taking into account mass transport limitations and slower diffusion of electrolyte through the micro-porous channel.[S3] The MOF modified electrode can be considered as a working electrode, modified by non-conducting crystals having isolated redox active species. However, due to the microporous MOF structure ions can diffuse through the crystals. Hence electrochemical process can be written as:

$MOF + nA^{-}(sol) - ne^{-} \rightarrow [\, nA^{-} \subset (MOF)^{n+}]$

Or

1) $MOF - ne^{-} \rightarrow (MOF)^{n+}$;
2) $nA^{-}(sol) + (MOF)^{n+} \rightarrow [\, nA^{-} \subset (MOF)^{n+}]$

where A- is the counter ion of an electrolyte solution. The sign "⊂" indicates supramolecular assembly involving host and guest (*i.e.* Guest ⊂ Host). If electron transfer is slower compared to diffusion of counter ion A^{-}, the rate of electron transfer becomes the rate determining step, while the rate of diffusion of A^{-} will not have any influence on the cyclic voltammogram. A similar situation can be seen at the low scan rate. However, when the rate of diffusion of A^{-} is slower (or not significantly fast compare to electron transfer), the rate of diffusion of A^{-} begins to influence the cyclic voltammogram.

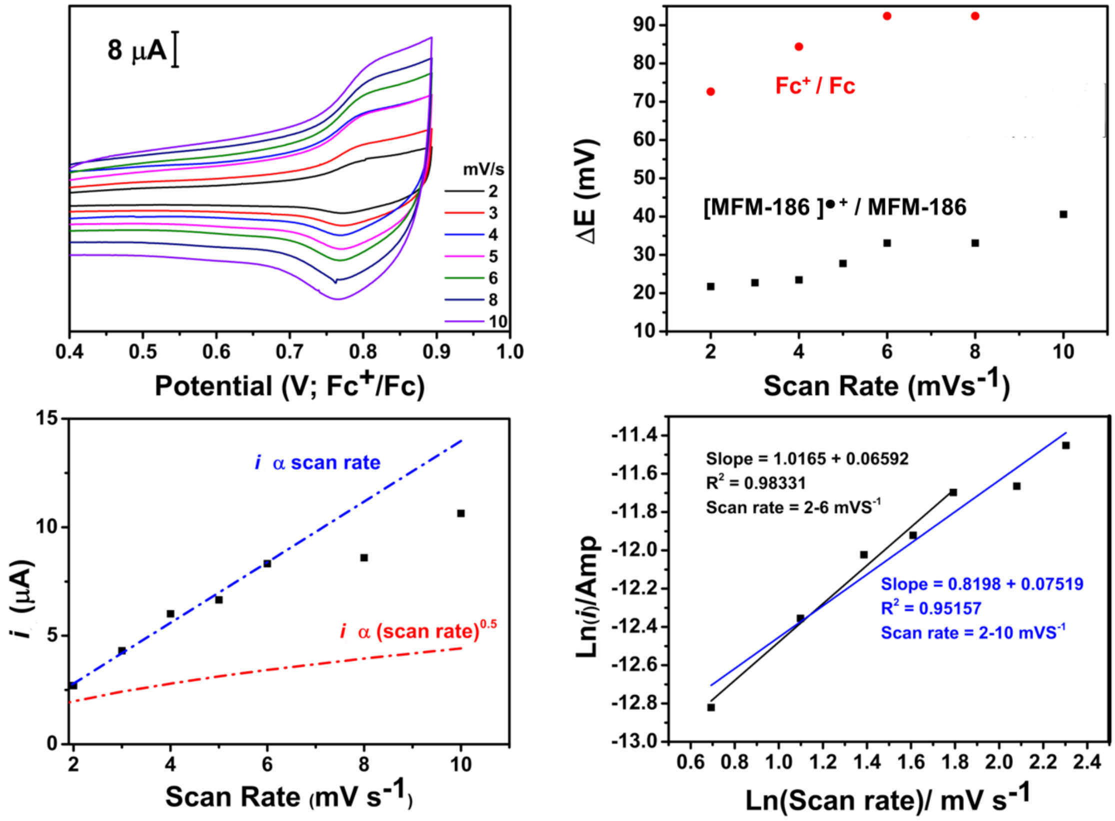

Figure S8: Cyclic voltammetry of MFM-186 film at various scan rates (a) and related analyses (b-d). Dependence of ΔE and i_p on scan rate strongly indicates that the electrochemical oxidation process of MFM-186 is not diffusion limited. This can be verified further by plotting Ln(i) *vs* Ln(scan rate); the slope is much higher than 0.5, as would be expected for a classical diffusion limited process. See preceding discussion for details.

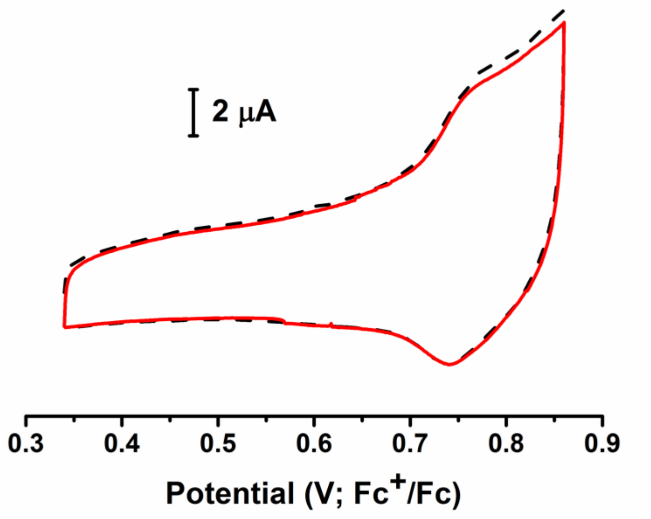

Figure S9: Multiple cycles in the cyclic voltammetric scan for MFM-186 films on carbon paper at scan rate 2 mV s^{-1}. The Broken line represents 1st scan while the solid line represents 3rd scan.

EPR spectroscopy of $[Et_4L^1]^{\bullet+}$

The experimental EPR spectrum of $[Et_4L^1]^{\bullet+}$ can be reasonably reproduced by simulation using hyperfine couplings to 3 sets of hydrogen nuclei (see parameters in Fig. S10). This suggests that the unpaired electron is localised mainly on the anthracene core, a result consistent with DFT analysis, although the smallest hyperfine couplings may indicate that some electron density extends to the adjacent phenyl rings.

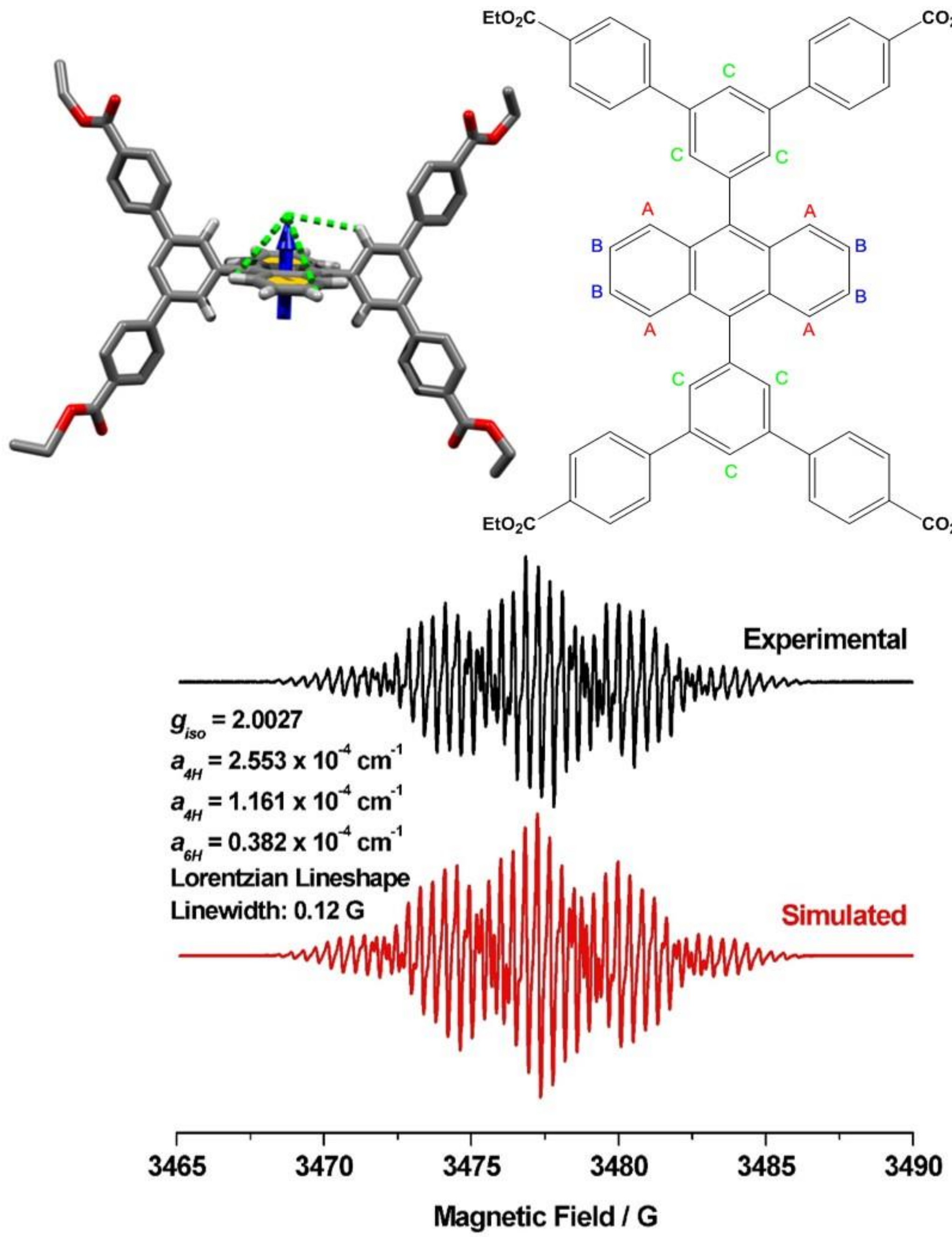

Figure S10: EPR spectrum of $[Et_4L^1]^{\bullet+}$ (bottom). Top: Hyperfine coupling to H centres in the EPR spectrum of $[Et_4L^1]^{\bullet+}$. Colour code: C; black, H; grey, O; red. Blue arrow indicates the position of the radical ion; green lines indicate 3 different hyperfine interactions. Three sets of different proton positions are highlighted as ‘A’, ‘B’ & ‘C’

Computational details: All DFT calculations were performed using the program Gaussian 03.[S4] Geometry optimizations were performed using the the Becke three-parameter hybrid exchange functional[S5] and the Lee–Yang–Parr correlation function[S6] (B3LYP) in combination with the the 6-31G(d,p)[S7] basis set for C, H and O atoms and the standard LANL2DZ basis set for the Cu atoms.[S8] After optimization a frequency analysis was performed to confirm that the stationary point was found to be a minimum on the potential energy surface. Molecular models were manipulated using the program Moldraw [S9] and visualization of optimized structure and plot of electronic properties were obtained with the program Molekel (version 5.4.0.8).[S10]

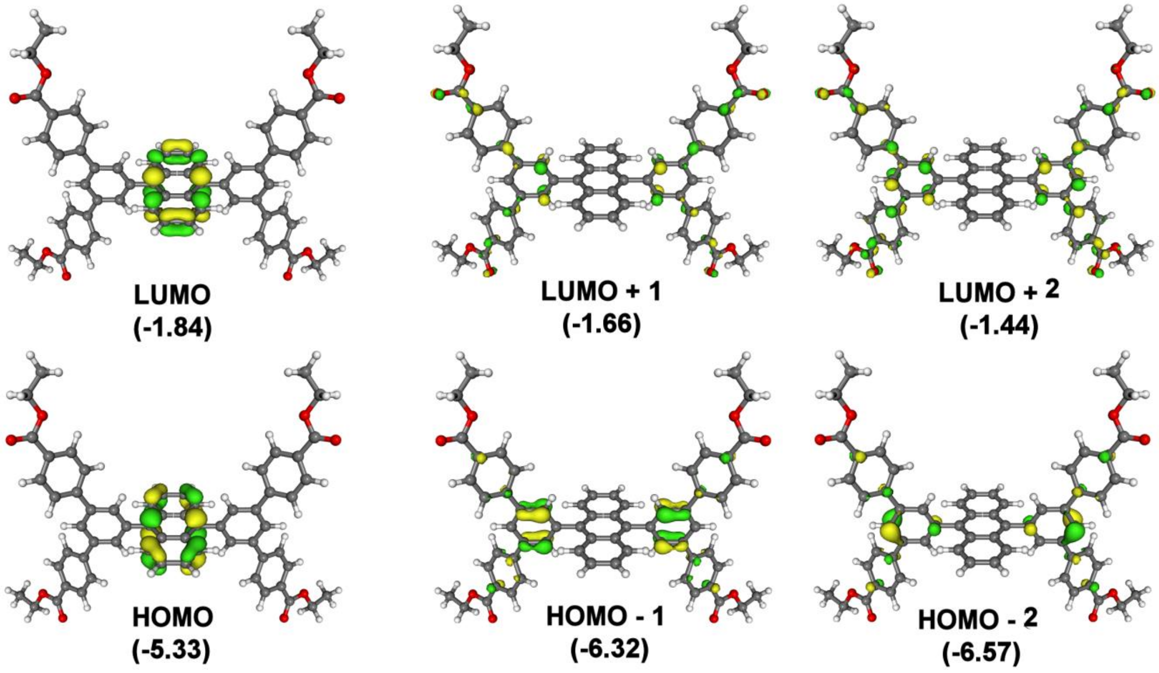

Figure S11: Frontier orbitals of Et_4L^1 and their energy (in eV against vacuum)

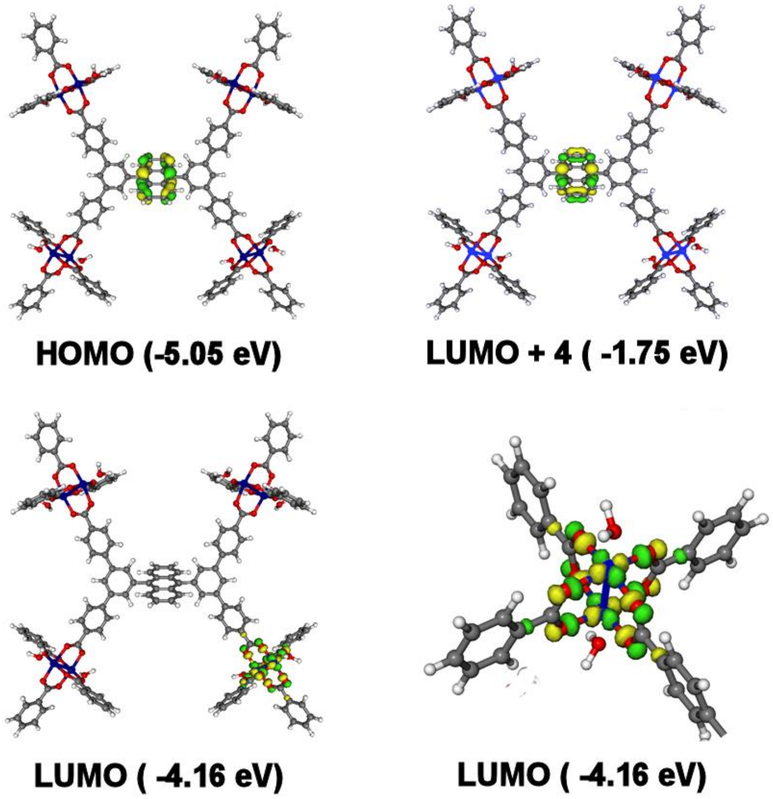

Figure S12: Frontier orbitals of model system for [MFM-186] and their energy (in eV against vacuum)

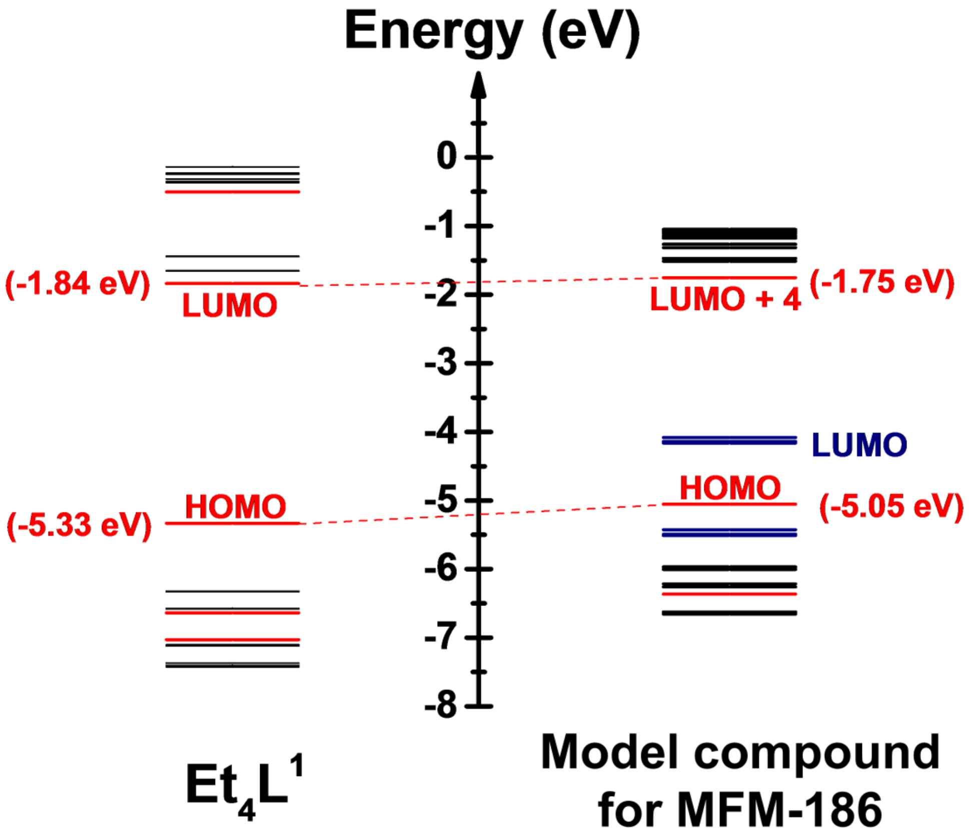

Figure S13: Comparison of the energy of frontier orbitals of Et_4L^1 and of the model system for [MFM-186]. The red levels indicate orbitals primarily confined to the anthracene motif, while blue levels show the Cu-paddlewheel-based orbitals. Note that the LUMO in the model system is Cu-paddlewheel-based which may explain delamination of MFM-186 films upon reduction.

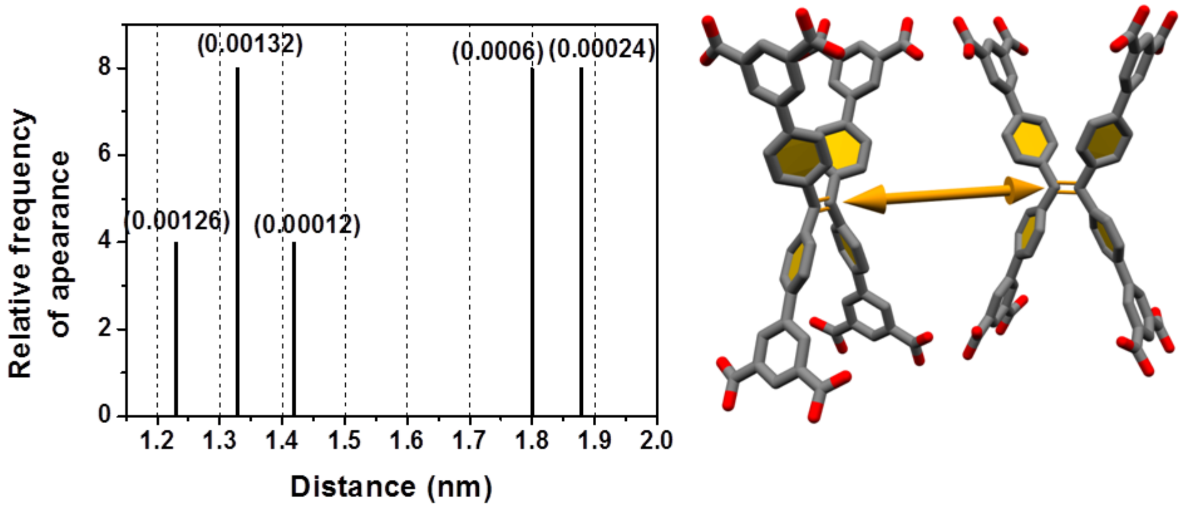

Figure S14: Interatomic distance between two ‘>C=C<’ groups in the MFM-180 framework, as seen in the single crystal structure. The number in parenthesis indicates error limit for distance.

S1. F. Moreau, D. I. Kolokolov, A. G. Stepanov, T. L. Easun, A. Dailly, W. Lewis, A. J. Blake, H. Nowell, M. J. Lennox, E. Besley, S. Yang and M. Schröder, *Proc. Nat. Acad. Sci (USA),* 2017, **114**, 3056-3061

S2. A. R. Chianese, A. Mo and D. Datta, *Organometallics*, 2009, **28**, 465-472.

S3. A. D. Carbó, Electrochemistry of Porous Materials, CRC Press, 2009.

S4. M. J. Frisch, G. W. Trucks, H. B. Schlegel, G. E. Scuseria, M. A. Robb, J. R. Cheeseman, J. Montgomery, J. A, T. Vreven, K. N. Kudin, J. C. Burant, J. M. Millam, S. S. Iyengar, J. Tomasi, V. Barone, B. Mennucci, M. Cossi, G. Scalmani, N. Rega, G. A. Petersson, H. Nakatsuji, M. Hada, M. Ehara, K. Toyota, R. Fukuda, J. Hasegawa, M. Ishida, T. Nakajima, Y. Honda, O. Kitao, H. Nakai, M. Klene, X. Li, J. E. Knox, H. P. Hratchian, J. B. Cross, V. Bakken, C. Adamo, J. Jaramillo, R. Gomperts, R. E. Stratmann, O. Yazyev, A. J. Austin, R. Cammi, C. Pomelli, J. W. Ochterski, P. Y. Ayala, K. Morokuma, G. A. Voth, P. Salvador, J. J. Dannenberg, V. G. Zakrzewski, S. Dapprich, A. D. Daniels, M. C. Strain, O. Farkas, D. K. Malick, A. D. Rabuck, K. Raghavachari, J. B. Foresman, J. V. Ortiz, Q. Cui, A. G. Baboul, S. Clifford, J. Cioslowski, B. B. Stefanov, G. Liu, A. Liashenko, P. Piskorz, I. Komaromi, R. L. Martin, D. J. Fox, T. Keith, M. A. Al-Laham, C. Y. Peng, A. Nanayakkara, M. Challacombe, P. M. W. Gill, B. Johnson, W. Chen, M. W. G. Wong, C. and J. A. Pople, Gaussian (version 3), Gaussian Inc., Pittsburgh, PA, 2004.

S5. A. D. Becke, *J. Chem. Phys.*, 1993, **98**, 5648-5652.

S6. C. Lee, W. Yang and R. G. Parr, *Phys. Rev. B*, 1988, **37**, 785-789.

S7. R. S. Grev and H. F. Schaefer, *J. Chem. Phys.*, 1989, **91**, 7305-7306.

S8. C. E. Check, T. O. Faust, J. M. Bailey, B. J. Wright, T. M. Gilbert and L. S. Sunderlin, *J. Phys. Chem. A*, 2001, **105**, 8111-8116.

S9. P. Ugliengo, D. Viterbo and G. Chiari, Z. Kristallogr. , 2010, **207**, 9-23.

S10. Molekel (version 5.4.0.8), Swiss National Supercomputing Center, 2006
